# Adductor Canal Block Combined with General Analgesia for Patients with Recurrent Patellar Dislocation Undergoing “3‐in‐1” Procedure Surgery: A Prospective Randomized Controlled Trial

**DOI:** 10.1111/os.13706

**Published:** 2023-05-16

**Authors:** Yan Xiong, Duan Wang, Shu Li, Xuejie Li, Yanjun Lin, Jian Li, Qi Li

**Affiliations:** ^1^ Department of Orthopaedics, Orthopedic Research Institute, West China Hospital Sichuan University Chengdu People's Republic of China; ^2^ Department of Anesthesiology West China Hospital of Sichuan University Chengdu People's Republic of China

**Keywords:** Adductor Canal Block, Analgesia, Patellar Dislocation, Ropivacaine, Saphenous Nerve

## Abstract

**Objective:**

Patellar dislocation is a common injury in sports medicine. While surgical treatment is an important option, pain is severe after surgery. This study compared the analgesic effect and early rehabilitation quality between adductor canal block combined with general analgesia (ACB + GA) and single general analgesia (SGA) after recurrent patellar dislocation (RPD) for “3‐in‐1” procedure surgery.

**Methods:**

From July 2018 to January 2020, a prospective randomized controlled trial was conducted in analgesia management after RPD for “3‐in‐1” procedure surgery. The 40 patients in the experimental group received ACB (0.3% ropivacaine 30 mL) + GA, while the 38 patients in the control group received SGA. Patients in both groups received “3‐in‐1” procedure surgery, standardized anesthesia, and analgesia during hospitalization. The outcomes included the visual analog scale (VAS), quadriceps strength, Inpatient Satisfaction Questionnaire (IPSQ), Lysholm scores, and Kujala scores. Total rescue analgesic consumption and adverse events were also recorded. One‐way analysis of variance (ANOVA) was used to compare continuous variables between groups and chi‐square or Fisher's exact tests were used to compare count data. Nonparametric Kruskal–Wallis H tests evaluated ranked data.

**Results:**

No significant differences in resting VAS scores were observed at 8, 12, and 24 h postoperatively. However, the flexion and moving VAS scores of the ACB + GA group were significantly lower than those of the SGA group (*p* < 0.05). Meanwhile, the first triggering of rescue analgesics was advanced in the SGA group (*p* < 0.0001), and the dose of opioid analgesics was significantly higher (*p* < 0.0001). The quadriceps strength of the ACB + GA group was higher than that of the SGA group at 8 h postoperatively. The IPSQ of the ACB + GA group was significantly higher 24 h postoperatively. We observed no significant differences in Lysholm and Kujala scores between the two groups at 3 months after surgery.

**Conclusions:**

Early analgesia management of ACB + GA showed excellent analgesia effectiveness and a positive hospitalization experience for RPD patients undergoing “3‐in‐1” procedure surgery. Moreover, this management was good for early rehabilitation.

## Graphical abstract

Postoperative pain of the “3‐in‐1” procedure surgery in RPD is acute. ACB + GA showed excellent analgesia effectiveness. Meanwhile, it was good for early rehabilitation. “3‐in‐1” procedure surgery with recurrent patellar dislocation and adductor canal block under ultrasound‐guidance. ① Lateral patellofemoral ligament release. ② Lower‐inner transfer of the tibial tuberosity. ③ Reconstruction of the medial patellofemoral ligament. ④ The operational procedure of adductor canal block:SA, sartorius; VM, vastus medialis; SN, saphenous nerve; FA, femoral artery.

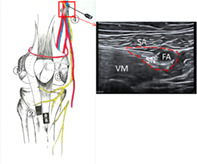

## Introduction

Patellar dislocation (PD) refers to inwards patellar prolapse or outwards femoral trochlea.^1^ PD is a common knee injury in children and adolescents, especially females. PD accounts for 3% of knee injuries and is mostly associated with bone dysplasia, ligamentous laxity, lower limb deformity, and heredity.^2^, ^3^, ^4^ The treatment options include conservative and operative methods. Conservative treatment is mostly used for initial acute traumatic PD. The reported probability of failure for conservative treatment is up to 70%; such failure can lead to recurrent patellar dislocation (RPD).^5^, ^6^ RPD tends to cause patellofemoral arthritis, persistent knee pain, quadriceps atrophy, etc. Operative management may be required in patients who experience recurrent episodes of dislocation. The treatment outcomes involve both the elimination of recurrent instability episodes and continued satisfactory patellar function.^5^, ^6^ The surgical goals for RPD are to reestablish an appropriate balance among the four‐quadrant cruciform forces acting on the patella. Surgical techniques, including releasing tight lateral structures, restoring medial restraints, and improving anatomic alignment proximally or distally to the patella, have been proposed.^5^, ^6^, ^7^, ^8^ The “3‐in‐1” surgical procedure, which combines lateral release, reconstruction of the medial patellofemoral ligament, and transfer of the tibial tuberosity in RPD, has demonstrated good clinical results and low patellar redislocation rates.^7^, ^8^

However, surgical trauma is increased for the “3‐in‐1” procedure in RPD. Meanwhile, the postoperative pain of RPD is acute, with visual analog scale (VAS) scores reaching 60 to 90 points.^9^ Thus, there is an urgent demand for postoperative analgesics. Pain, especially early postoperative pain, can seriously affect postoperative limb function rehabilitation and can easily lead to physical stress reactions and a continuous release of inflammatory factors.^10^ Analgesia management after knee surgery has been reported in knee arthroplasty, knee arthroscopy, and knee ligament reconstruction.^11^, ^12^, ^13^ The commonly used analgesic schemes include general analgesia (GA), peripheral nerve block, and local or joint cavity injection analgesia.^11^, ^12^, ^13^, ^14^ GA, especially opioid analgesics, has certain side effects on other organs. Meanwhile, regional peripheral nerve block analgesia has become a hot focus in recent years. After knee operations, common local block analgesics, including femoral nerve block, adductor canal block (ACB), and sciatic nerve block, are performed. Studies on analgesic effects after knee arthroplasty showed similar clinical outcomes between femoral nerve block and ACB anesthesia. However, femoral nerve block affected quadriceps strength, which was not helpful for early rehabilitation training.^13^, ^14^ The literature had confirmed that adductor canal saphenous nerve block was a safe and effective analgesic method for knee surgery, which also improved quadriceps strength. Patients underwent early activity, reducing postoperative complications and promoting functional rehabilitation.^13^, ^14^ We hypothesized that ACB + GA could effectively reduce postoperative pain of RPD, and promote early rehabilitation. Therefore, a prospective clinical randomized controlled trial of analgesia management of ropivacaine for ACB + GA was conducted to observe and analyze the early analgesic effects and quality of rehabilitation after surgical treatment of RPD.

## Materials and Methods

### The Clinical Trial Design

**Fig. 1:**
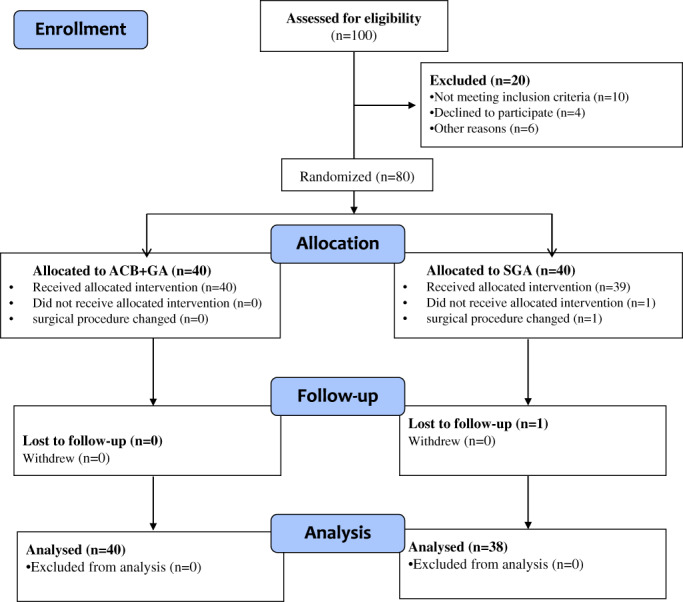
Consolidated Standards of Reporting Trial (CONSORT) diagram of patient flow through the study

From July 2018 to January 2020, a prospective randomized controlled trial of analgesia management using ropivacaine for ACB + GA after “3‐in‐1” procedure surgery for RPD was conducted at the Department of Sports Medicine, West China Hospital of Sichuan University. All procedures described in this study were registered with the Chinese Clinical Trial Registry (ChiCTR2000032484) and approved by Biomedical Ethics Committee, West China Hospital of Sichuan University (No. 2018‐034). Inclusion criteria were as follows: 14–60 years old, no gender limitation, growth plate closed, American Society of Anesthesiologists (ASA) physical classification I to II, body mass index (BMI) less than 35 kg/m^2^, RPD with TT‐TG>20, Caton>1.2, patellar tilt angle>20°, not associated with obvious bone deformity of lower limb, and undergoing three‐in‐one surgery. Exclusion criteria included contraindications for ACB nerve block, allergy or intolerance to trial medications, a previous surgical history on the operative limb, existing neuropathy on the operative limb, intraoperative changes in surgical methods, and refusal of postoperative follow‐up. The experimental group was ropivacaine for ACB + GA, and the control group was SGA. The computer randomly generated sequences, which were sealed in envelopes and opened by research assistants before anesthesia to ensure that all researchers and patients were unaware. All operations were performed by a senior surgeon team (Pro. Jian Li). All included patients signed an informed consent form (Figure 1).

### Anesthesia and Operation Procedures

**Fig. 2:**
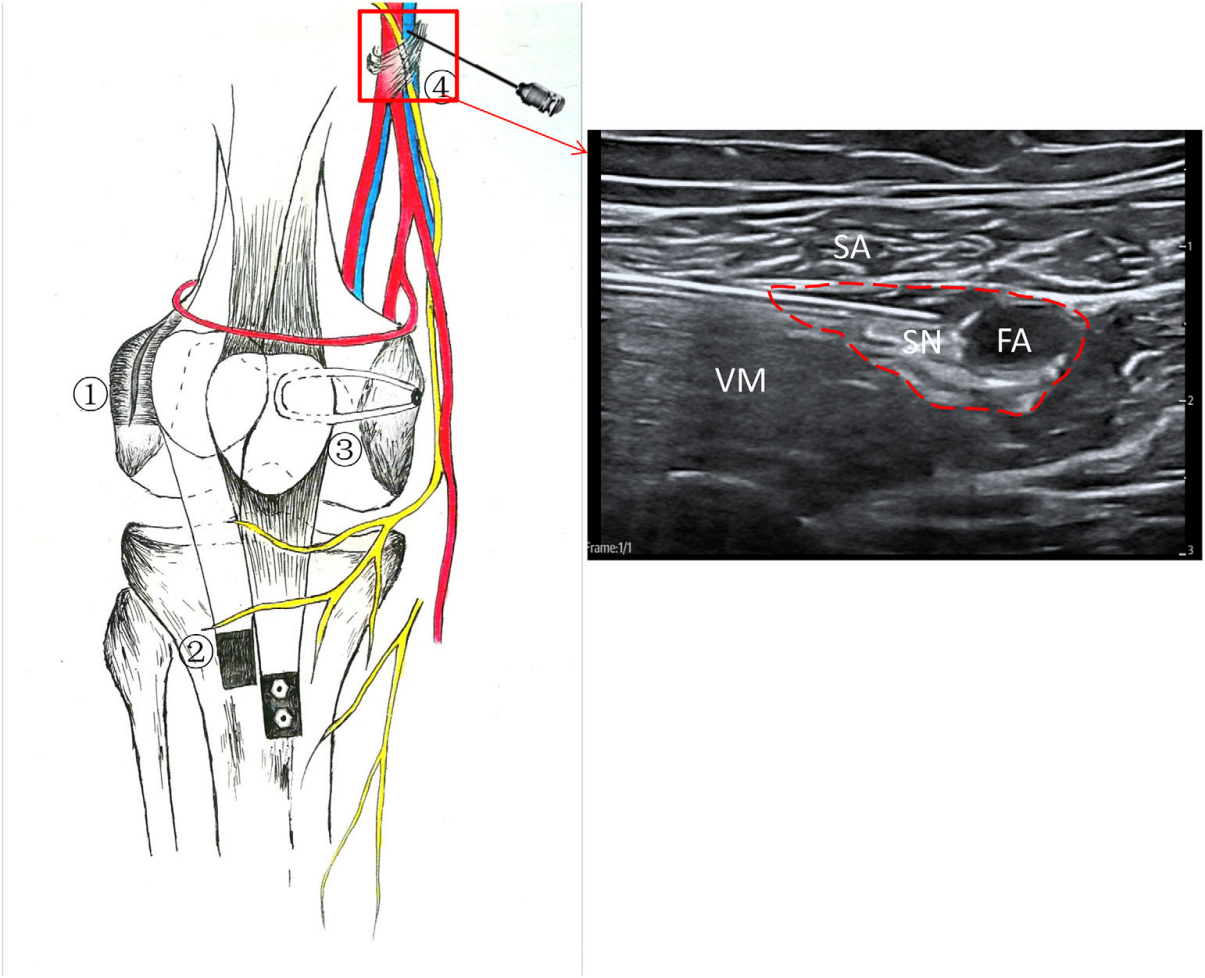
“3‐in‐1” procedure surgery with recurrent patellar dislocation and adductor canal block under ultrasound‐guidance. ① Lateral patellofemoral ligament release. ② Lower‐inner transfer of the tibial tuberosity. ③ Reconstruction of the medial patellofemoral ligament. ④ The operational procedure of adductor canal block. SA, sartorius; VM, vastus medialis; SN, saphenous nerve; FA, femoral artery

Anesthesia was induced before surgery. After anesthesia was performed, the experimental group received an ACB nerve block using 0.3% ropivacaine 30 mL (AstraZeneca AB, Sweden) under ultrasound guidance (GE, USA) by a senior anesthesia team. The level of the nerve block was the midpoint of the line between the anterior superior iliac spine and the knee joint (Figure 2). The two groups of patients underwent knee arthroscopy to observe the patellofemoral movement trajectory and repair the damaged cartilage. Eight patients had loose bodies removed from the knee. After arthroscopy, an arc‐shaped incision was made along the anterior medial of the knee joint from the upper edge of the patella to the tibial tuberosity. The semitendinosus tendon from female patients was harvested for reconstruction of the medial patellar ligament while the gracilis tendon was harvested from male patients, with a required diameter of 3 to 3.5 mm. Tibial tuberosity transfer was performed and the tense lateral retinaculum of the patella was loosened. The tibial tuberosity transfer was based on a Caton–Deschamps index of 0.6–1.0, TT‐TG‐distance <20 mm, and completely extended patellar ligament without tension with the knee straight. The shifted tibial tuberosity was fixed using two absorbable polylactic acid screws (ConMed Linvatec Biomaterials Ltd., Finland). The patellar tunnel was established at the midpoint and upper midpoint of the inner edge of the patella, while a femoral bone tunnel was established at the anatomic termination point of the medial patellofemoral ligament (MPFL). The graft tendon was fixed tightly at 90° of knee flexion. Knee flexion and extension tests were then performed over the full range. The patella was in a good position and had a good range of motion. In addition, it showed no patellar dislocation in either the inwards to outwards tests. Ethibond sutures were used to secure the injured MPFL and the anatomic termination point of the pes anserinus tendon. Other structures and tissues were sutured in layers and arthroscopy was routinely performed before the skin was sutured to further evaluate the patellofemoral movement trajectory (Figure 2).^15^

### Postoperative Analgesic Scheme

The conventional general analgesic scheme was an intramuscular injection of nonsteroidal anti‐inflammatory drugs (NSAIDs) (parecoxib sodium 40 mg for adults over 18 years of age or flurbiprofen axetil injection 50 mg for patients under 18 years old qualer, 12 h per day) postoperatively in the two groups of patients (parecoxib sodium, Pfizer, USA; flurbiprofen axetil injection, TIDE, China). If the patient's resting pain index (visual analog scale [VAS]) was above 4 points, temporary analgesia was administered. The first administration was an intramuscular injection of tramadol hydrochloride 100 mg (Grunenthal GmbH, Germany). The second administration was an intramuscular injection of pethidine hydrochloride 50 mg (Qinghai, China), with multiple administrations not exceeding 150 mg within 24 h.

### Postoperative Rehabilitation Program

Elastic bandages were applied at the operative site for pressure bandaging and cold therapy was performed after the operation. Immediately after waking up from anesthesia, ankle pump motion and quadriceps contraction training began. On the first postoperative day, a full weight‐bearing walking exercise was performed by a knee brace locked in extension. Range of motion exercise with no external force began. All patients met the discharge criteria within 72 h postoperatively. At 4 weeks, the knee flexed to 90°. At 6 weeks, the knee flexed more than 120°. At 8 weeks, the patients removed the knee brace and walked normally. At 12 weeks, the patients returned to daily activities.

### Data Collection and Statistical Analysis

VAS scores at rest and quadriceps strength were evaluated at 2, 8, 12, 24, 48, and 72 h postoperatively. VAS scores during activity (flexion and moving) and Inpatient Satisfaction Questionnaire (IPSQ) scores were evaluated at 24, 48, and 72 h postoperatively.^16^ The types and total usage of analgesics were recorded 24 h after the operation. The Lysholm knee score and Kujala score were recorded preoperatively and 3 months postoperatively. The recorded complications included adverse events related to ACB (puncture site bleeding, hematoma, nerve damage, etc.), cardiovascular adverse events (hypotension, bradycardia, etc.), as well as the occurrence of nausea and vomiting, wound healing, knee joint effusion, lower limb swelling, and venous thrombus. According to the Modified Ashworth scale (MAS), the quadriceps strength (level 0–5) of the injured limb was measured while being protected by a knee brace set to 0°.^12^ All data were observed by a nonsurgery nurse with a blinded method. All analyses were performed using SPSS Statistics for Windows, version 20.0 (IBM, USA). Measurement data were expressed as the means ± standard deviations (x ± s). One‐way analysis of variance (ANOVA) was used for comparisons between groups, while least significant difference (LSD) tests were used for pairwise comparisons. Chi‐square or Fisher's exact tests were used to compare count data and nonparametric Kruskal–Wallis H tests were used to evaluate ranked data. *p* < 0.05 was considered statistically significant.

## Results

### Brief Information on Patients

A total of 100 patients with RPD were enrolled, of whom 80 met the inclusion criteria. According to random selection, 40 and 38 patients had received ACB + GA and SGA respectively, for “3‐in‐1” operations and completed follow‐up (Figure 1). The patients in the ACB + GA group were aged 15–33 years (mean 20.9 ± 4.8 years). The patients in the SGA group were aged 17–27 years (mean 22.1 ± 5.7 years). There were no statistically significant differences in the preoperative age, sex, body mass index (BMI), American Society of Anesthesiologists (ASA) physical status classification, number of dislocations, cartilage injuries, or other general conditions between these two groups. The quadriceps femoris strength of the operative limb was 5 and VAS was 0 in the two groups (Table 1).

**TABLE 1:** Patient demographic characteristics and duration of the surgical procedure values are given as the median ± standard deviation *, number of patients (%) ^**&**^, median with the interquartile range in parentheses

	ACB + GA (n = 40)	SGA (n = 38)	*p* Value
Age*(yr)	20.9 ± 4.8	22.1 ± 5.7	0.292
Gender (Male/Female)	14/26	11/27	0.632
BMI* (kg/m^2^)	21.1 ± 1.5	21.1 ± 1.8	0.912
Side of knee (right/left)	21/19	17/21	0.507
Number of dislocations^§^	4 (3.3, 6.0)	4 (3.0, 5.0)	0.396
cartilage injuries ≥ gr 2^&^	13 (32.5%)	11 (28.9%)	0.734
TT‐TG‐distance (mm)*	23.2 ± 1.3	22.9 ± 1.3	0.349
Caton–Deschamps index*	1.37 ± 0.04	1.35 ± 0.04	0.565
ASA physical status, I/II	40/0	38/0	n.s
Duration of surgery* (min)	53.1 ± 5.3	52.1 ± 5.9	0.422

*Note*: Cartilage injuries using Kellgren‐Lawrence criteria.

Abbreviations: ACB, adductor canal block; ASA, American Society of Anesthesiologists; BMI, Body mass index; SGA, single general analgesia; TT‐TG‐distance, Tibial Tuberosity‐Trochlear Groove Distance.

### General Information on Anesthesia and Operation

Patients in both groups successfully completed the “3‐in‐1” procedure surgery for RPD. The operation times ranged from 45 to 65 min (mean 53.1 ± 5.3 min) in the ACB + GA group and from 45 to 65 min (mean 52.1 ± 5.9 min) in the SGA group. There were no significant differences in the duration of anesthesia or operation time between the two groups (*p* < 0.05) (Table 1). Likewise, no significant differences were observed in the dosages of propofol, sufentanil, and remifentanil during the operation (*p* > 0.05). The patients' vital signs in both groups were stable, with no fever, palpitation, breathlessness, or other symptoms of discomfort. Ten percent (4/40) of patients in the ACB + GA group experienced analgesic side effects (two experienced nausea, one experienced dizziness, and one experienced pruritus) after surgery, compared to 13% (5/38) in the SGA group (three patients with nausea, one with dizziness, and one with pruritus), which improved after conventional treatment. No adverse events related to ACB such as nerve injury, puncture site bleeding, hematoma, and infection were observed in the ACB + GA group (Table 2).

**Table 2:** Analgesic dosage and drug‐related side effects in two groups

	ACB + GA		SGA		
	No. of patient	Mean	No. of patient	Mean	*p*
NSAIDs					
Parecoxib	30	60 ± 35.1	30	63.3 ± 33.2	0.684
Flurbiprofen Axetil	10	22.5 ± 40.7	8	21.1 ± 41.3	0.877
Tramadol	20	50 ± 49.0	27	100 ± 77.1	0.001
Opioid Pethidine	1	1.3 ± 7.9	7	9.2 ± 19.6	0.02
Nausea	2	‐	3	‐	n.s
Dizziness	1	‐	1	‐	n.s
Pruritus	1	‐	1	‐	n.s

*Note*: Values are given as the median ± standard deviation.

Abbreviations: ACB, adductor canal block; SGA, single general analgesia.

### VAS Scores

**Fig. 3:**
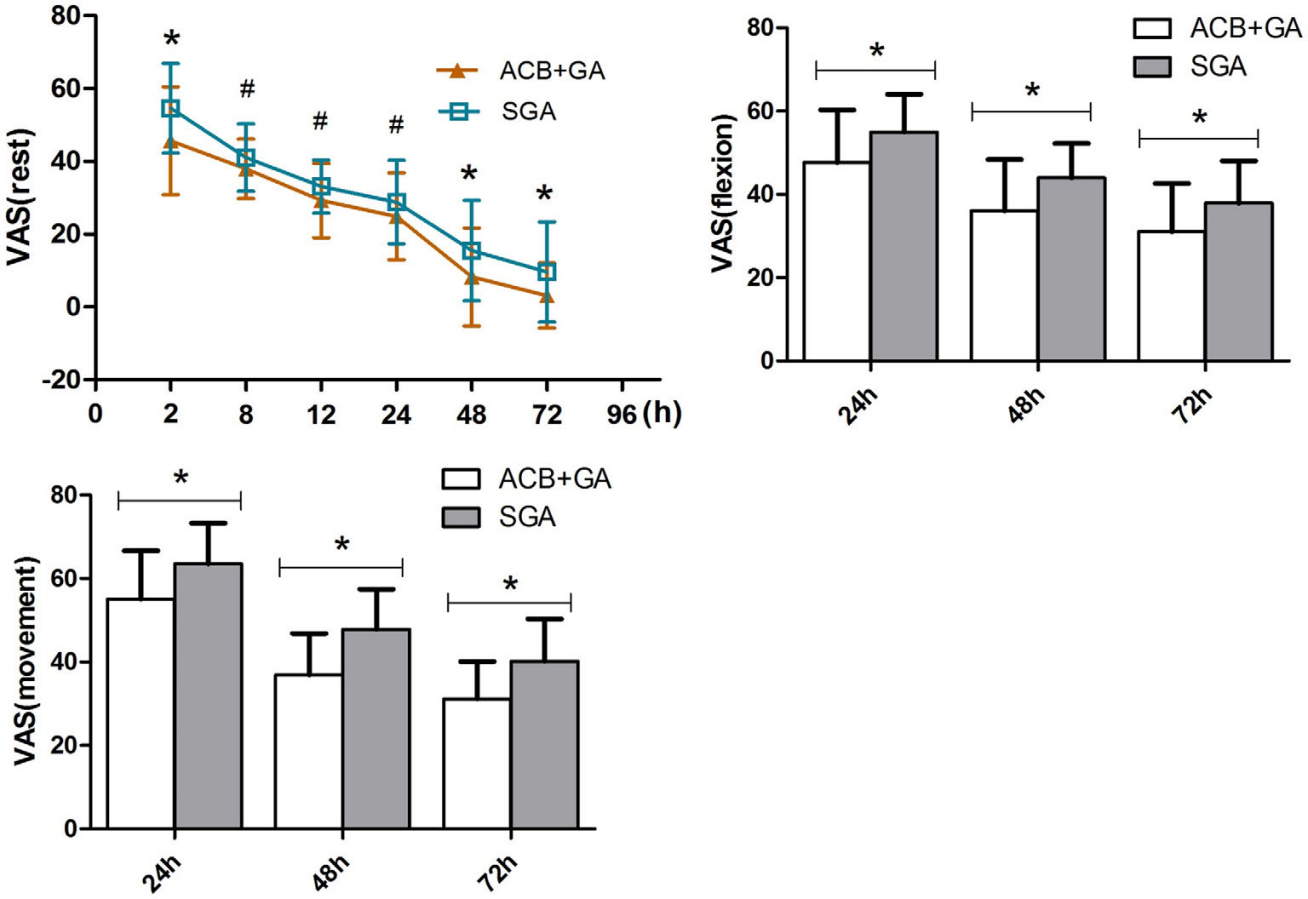
VAS scores at rest and activity at 2 h, 8 h, 12 h, 24 h, 48 h, and 72 h after operation. VAS, sisual analog scale (VAS; with 0, no pain, to 100, the worst imaginable pain), ACB, adductor canal block, GA, general analgesia, SGA, single general analgesia. ACB + GA group vs. SGA group by one‐way ANOVA test. (A) *P_*2,48,72h*_ *= 0.013*, 0.027, 0.017, ^*#*^ *P*_*8,12*_,_*24h*_ = 0.123, 0.068, 0.142. (B) *P_*24*_,_*48*_,_*72h*_ = 0.004, 0.001, 0.006. (C) *P_*24*_,_*48*_,_*72h*_ = 0.001, 0.000, 0.000

Postoperatively, the VAS scores increased significantly in both groups, reaching up to 70–90 points within 24 h. At rest, the VAS pain scores of the ACB + GA group were significantly lower at 2 h postoperatively than those of the SGA group (*p* = 0.013). Due to the good postoperative analgesia management in the two groups, there were no significant differences in VAS scores at 8, 12, and 24 h (*p* > 0.05). However, the first triggering rescue analgesics occurred significantly earlier in the SGA group (95% confidence interval [CI], 0.12 to 0.39; *p* < 0.0001). Both had pain relief after temporary analgesic administration. The differences in VAS scores between the two groups of patients were more significant during activity at 24, 48, and 72 h (*p* < 0.05). With early rehabilitation training and persistent pain, the VAS pain scores at rest in the SGA group were significantly higher at 48 and 72 h than in the ACB + GA group (*p* = 0.027, 0.017) (Figure 3).

### Quadriceps Strength and IPSQ Score

**Fig. 4:**
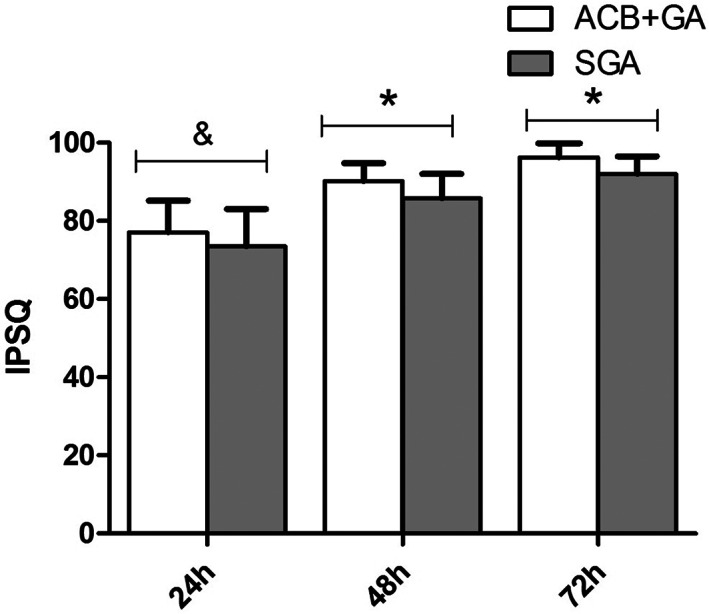
Inpatient Satisfaction Questionnaire at 24 h, 48 h, and 72 h after operation. IPSQ, Inpatient Satisfaction Questionnaire (0 to 100 score), ACB, adductor canal block, GA, general analgesia, SGA, single general analgesia. ACB + GA group vs. SGA group by one‐way ANOVA test. ^*&*^ *P*_*24h*_ = 0.081. *P_*48*_,_*72h*_ = 0.001, 0.000

Because of surgical trauma and pain stress, most patients in the two groups could not lift their leg off the bed at 2 h postoperatively, with no significant difference between groups (*p* = 0.093). At 24 h after surgery, the quadriceps strength of all patients in the ACB + GA group reached level 4 and more and they could walk with the protection of a knee brace. Meanwhile, in the SGA group, only 63% (24/38) of patients showed quadriceps strength reaching level 3 and reached level 4 after more than 72 h. Significant differences in quadriceps strength were observed between the groups at 8, 12, 24, 48, and 72 h (*p =* 0.020, <0.0001, <0.0001, <0.0001, and 0.041, respectively). Therefore, there were also differences between the two groups in the process of rehabilitation training, such as leg lifting and walking. Due to pain irritation, multiple analgesic rescue administrations, and slower quadriceps muscle strength recovery, the IPSQ scores of the SGA group were significantly lower at 24 h postoperatively than those in the ACB + GA group (Figure 4).

### Extra Analgesic Usage

**Fig. 5:**
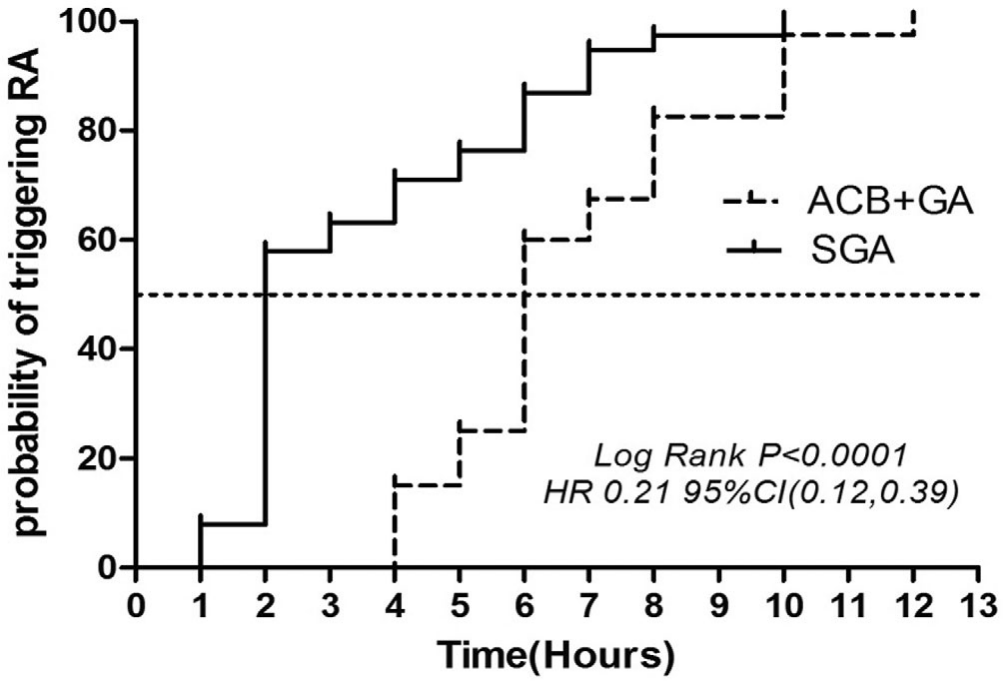
Kaplan–Meier curve of time required for the first rescue analgesics. RA, rescue analgesics. ACB, adductor canal block, GA, general analgesia, SGA, single general analgesia

Significant differences in the frequencies and dosages of postoperative analgesics were observed between the two groups of patients. In the SGA group, the requirement for the first analgesic occurred significantly earlier (95% CI, 0.12 to 0.39; *p* < 0.0001), with 58% (22/38) of patients starting supplemental analgesics within 2 h postoperatively, which increased to 100% using at 10 h postoperatively (Figure 5). Moreover, 29% (11/38) of patients in the SGA group received additional analgesics twice within 24 h after the operation, and 18% (7/38) were treated with dolantin for analgesia. In the ACB + GA group, only one patient was administered supplemental dolantin within 24 h postoperatively. The 24‐h dosages of tramadol and dolantin were significantly lower in the ACB + GA group than in the SGA group (*p* = 0.001 and 0.020, respectively) (Table 2).

### Knee Function

Knee joint function recovered well, with the patella stable and no redislocation, and negative extrapolation apprehension tests in both groups. In the ACB + GA group, at the 3‐month follow‐up, the Lysholm and Kujala scores had increased from 50.5 ± 6.5 and 48.3 ± 5.6, respectively, before the operation to 87.7 ± 2.3 and 85.8 ± 2.5. Similarly, in the SGA group, the Lysholm and Kujala scores increased from 50.7 ± 5.0 and 48.0 ± 3.0 before the operation to 87.2 ± 2.2 and 87.2 ± 2.2, respectively, with no significant differences between the two groups (*p* > 0.05) (Table 3).

**TABLE 3:** The functional rehabilitation in two groups

	Lysholm			Kulaja		
	Pre	1m	3m	Pre	1m	3m
ACB + GA	50.5 ± 6.5	69.1 ± 5.1	87.7 ± 2.3	48.3 ± 5.6	68.8 ± 4.6	85.8 ± 2.5
SGA	50.7 ± 5.0	67.4 ± 3.8	87.2 + 2.2	48.0 ± 3.0	67.4 ± 3.8	87.2 ± 2.2
*p*	0.842	0.106	0.312	0.817	0.144	0.013

*Note*: Values are given as the median ± standard deviation.

Abbreviations: ACB, adductor canal block; SGA, single general analgesia.

### Complications

No limb swelling occurred in the ACB + GA group after surgery and vascular ultrasound examination showed no venous thrombus of the lower limbs 24 h postoperatively. In the SGA group, 24 h after the operation, four cases experienced crus swelling of different degrees. The patients were instructed to elevate the affected limbs and performed ankle pump exercises to reduce edema. One case of tension vesicle was observed. Skin vesicle was punctured and healed after wound dressing. Two cases of intermuscular venous thrombus were observed on ultrasound examination. Low molecular weight heparin (Sanofi, France) were injected (0.4 ml, q 12 h), and venous thrombosis were confirmed to disappear by ultrasound reexamination before discharge. Neither group had wound infection. Only one patient in the ACB + GA group had fat liquefaction and the wound healed after a fresh dressing change. One patient in the SGA group suffered from knee stiffness. Knee ROM returned to normal under manipulation under anesthesia (MUA) at 6 weeks postoperatively.

## Discussion

In this study, the most important finding was that analgesia management of ACB + GA could effectively reduce postoperative pain of RPD, and promote early rehabilitation. Especially the VAS pain scores at rest and activity in the ACB + GA group were significantly lower at 48 and 72 h after the operation than in the GA group. Meanwhile, the frequencies and dosages of postoperative analgesics were significantly lower in the ACB + GA group. Quadriceps strength and IPSQ scores were higher in the ACB + GA group after the operation. The process of rehabilitation training was effectively improved in the ACB + GA group. There were no significant differences in Lysholm and Kujala scores between the two groups at 3 months after surgery.

### Different Analgesic Methods Compare

The American Pain Society defined pain as the fifth vital sign.^17^ Pain not only causes patient anxiety and dysphoria but also affects postoperative rehabilitation.^9^, ^17^ Therefore, the management of perioperative analgesia in orthopaedics has been the focus of clinical research. Analgesia management after knee surgery has been widely used in fracture, knee arthroplasty, and anterior cruciate ligament (ACL) reconstruction.^12^, ^14^ The commonly used analgesic schemes include GA, peripheral nerve block, cocktail therapy, and joint cavity injection analgesia.^11^, ^12^, ^13^, ^14^ GA often uses intravenous analgesia, mostly opioids. However, the risk of adverse reactions to opioids cannot be ignored in clinical practice. These reactions mainly include respiratory function inhibition, gastrointestinal dysfunction, nausea, vomiting, headache, dizziness, and even addiction, which may cause drug dependence.^18^, ^19^ ACB and femoral nerve block can also effectively relieve postoperative pain and reduce analgesic consumption.^12^, ^14^, ^17^, ^18^ However, femoral nerve block reportedly reduced quadriceps muscle strength, increased the risk of falls, and delayed rehabilitation.^12^, ^13^, ^14^ Additionally, periarticular injection also effectively control pain combined with peripheral nerve block.^18^, ^19^, ^20^ In the present trial, early analgesia management of ACB provided excellent analgesic effectiveness. Simultaneously, GA with NSAIDs (parecoxib sodium or flurbiprofen axetil injection) was used to supplemental analgesic efficacy, especially to provide anti‐inflammatory effects. Thus, the 24‐h dosages of tramadol and dolantin were significantly lower in the ACB + GA group. Due to the potential for neurovascular injury, hospitalization costs, and patient inconvenience, this study did not integrate a perineural catheter.

### Pain Release on ACB

Among the various surgical treatments for RPD, the “3‐in‐1” procedure is a proven surgical method.^7^, ^8^ The operation area of RPD is mainly the front and medial area of the knee joint, including the areas for anterior medial skin incision, hamstring tendon extraction, medial patellofemoral ligament reconstruction, and patellar ligament and tibial tuberosity osteotomy, which is mainly saphenous nerve innervation.^7^, ^8^, ^21^, ^22^ The adductor canal is a myofascial cavity containing the saphenous nerve, medial femoral cutaneous nerve, cutaneous branches of the obturator nerve, etc. The analgesic effect of the adductor canal saphenous nerve block in the ACB + GA group in this study was very obvious and can greatly relieve pain after recurrent patellar dislocation operation. However, a small number of patients respond poorly to ACB analgesia, which may be related to saphenous nerve variation, analgesic drug tolerance, low pain threshold, and lateral surgical area pain. Regarding acute pain caused by knee operation trauma, the pain is severe in the first 24 h and levels of inflammatory factors increase sharply.^13^, ^14^ GA with NSAIDs has anti‐inflammatory and supplemental analgesic effects on the above factors. The results of the present trial were consistent with previous reports. Previous studies recommended performing ACB 30 min before the operation for better preemptive analgesia in local tissue and to effectively reduce the trauma response of the body tissue at the beginning of the operation. An ACB plane at the mid‐thigh level, approximately 10–15 cm above the knee joint, has also been suggested as an accurate block for the saphenous nerve, with some reports of also blocking the medial femoral cutaneous nerve.^12^, ^14^, ^18^ Analgesia using ropivacaine for a saphenous nerve block is effective for more than 17 h, thus showing a good effect on early pain.^18^ However, there was no consensus regarding the optimal ropivacaine concentration and volume for ACB in previous studies.^12^, ^13^, ^14^, ^15^, ^18^, ^19^ In the present trial, the influence on rehabilitation after 24 h was persistent due to the good analgesic effect of the ACB. With early rehabilitation training and persistent pain, the VAS scores at rest and during moving, and flexion in the ACB + GA group were significantly lower at 24 h postoperatively. Meanwhile, the ACB + GA group showed reduced analgesic consumption and a significantly delayed time to first analgesic use.

### Rehabilitation Promotion of ACB

The concept of enhanced recovery after surgery (ERAS) has gained popularity. Without effective perioperative analgesia, ERAS cannot be applied.^23^, ^24^ Studies on ACB showed that the quadriceps strength after knee surgery was less affected. Thus, early functional training can be performed.^13^, ^18^ Moreover, due to muscle use, blood flow is increased, effectively reducing tissue edema and venous thrombus of the lower limbs and further speeding rehabilitation.^25^, ^26^, ^27^ The findings of the present study were consistent with these previous reports. After the “3‐in‐1” procedure for RPD, the ACB combined with GA controlled the pain score during activity to 40 or below. Patients can recover rapidly after surgery. Ankle pumping and quadriceps contraction training can be performed immediately after the operation, with walking training with knee brace protection performed on the first postoperative day and the knee flexed to 60°–90° in the Semi‐Fowler's position, all of which can effectively avoid quadriceps atrophy and knee adhesion after the operation. In this study, early analgesia management of ACB + GA provided pain relief and preserved the strength of the quadriceps femoris after the operation. The quadriceps strength of the ACB + GA group reached level 4 and more at 24 h postoperatively and was beneficial for early rehabilitation training. Moreover, the IPSQ score was high.

## Strengths and Limitations

The strength of this study was focusing on analgesia management after surgical treatment of RPD. ACB is a safety and reliable management. The analgesia management of ACB + GA showed excellent pain release and was good for early rehabilitation. Moreover, this study is an RCT, and the evidence stage is level I. The limitations were as follows: First, this trial was a small‐sample, single‐center, preliminary exploratory trial. Further multicenter studies with larger sample sizes are required. Second, the type, concentration, and volume of analgesics were relatively simple and subgroup analysis was not performed in the ACB + GA group. Third, due to the patellar ligament and quadriceps tendon operation for recurrent patellar dislocation, the protocol to measure quadriceps strength in this trial could not accurately evaluate the strength in the early postoperative period. Despite these limitations, the findings in this study deserve attention.

### Conclusions

The analgesia management of ACB + GA for RPD after the “3‐in‐1” procedure was easy to operate and safe. It showed excellent pain release, particularly at the flexion and moving state. Meanwhile, the first triggering of rescue analgesics was delayed, and opioid consumption was significantly declined. ACB + GA had little effect on the strength of the quadriceps femoris, and was good for early rehabilitation. Thus, it is worthy of clinical application.

## Author Contributions

Yan Xiong and Duan Wang participated in the collection of clinical data, performed patient follow‐ups, and drafted the paper. Shu Li collected the clinical data. Xuejie Li and Yanjun Lin were responsible for analgesia management. Jian Li performed operation. Qi Li made substantial contributions to conception and design of this research and has reviewed the paper for important intellectual content and given final approval of the version to be published. Each author has participated sufficiently in this work to take public responsibility for the appropriate portions of the paper. All authors read and approved the final paper.

## Conflicts of Interest

All authors have no conflicts of interest to report.

## Ethical Approval

All participants gave their written consent and were recruited in agreement with the Helsinki Declaration and approved by the Biomedical Ethics Committee (ID:2018‐034).

## Informed Consent

All included patients signed informed consent form. The informed consent is found in the supplementary material.
